# Development of a High Throughput Platform for Screening Glycoside Hydrolases Based on Oxime-NIMS

**DOI:** 10.3389/fbioe.2015.00153

**Published:** 2015-10-13

**Authors:** Kai Deng, Joel M. Guenther, Jian Gao, Benjamin P. Bowen, Huu Tran, Vimalier Reyes-Ortiz, Xiaoliang Cheng, Noppadon Sathitsuksanoh, Richard Heins, Taichi E. Takasuka, Lai F. Bergeman, Henrik Geertz-Hansen, Samuel Deutsch, Dominique Loqué, Kenneth L. Sale, Blake A. Simmons, Paul D. Adams, Anup K. Singh, Brian G. Fox, Trent R. Northen

**Affiliations:** ^1^US Department of Energy Joint BioEnergy Institute, Emeryville, CA, USA; ^2^Sandia National Laboratories, Livermore, CA, USA; ^3^Lawrence Berkeley National Laboratory, Berkeley, CA, USA; ^4^US Department of Energy Great Lakes Bioenergy Research Center, University of Wisconsin, Madison, WI, USA; ^5^Joint Genome Institute, Walnut Creek, CA, USA; ^6^Department of Bioengineering, University of California Berkeley, Berkeley, CA, USA; ^7^Department of Biochemistry, University of Wisconsin, Madison, WI, USA

**Keywords:** cellulase, NIMS, oxime bioconjugation, high throughput screening, enzyme assays

## Abstract

Cost-effective hydrolysis of biomass into sugars for biofuel production requires high-performance low-cost glycoside hydrolase (GH) cocktails that are active under demanding process conditions. Improving the performance of GH cocktails depends on knowledge of many critical parameters, including individual enzyme stabilities, optimal reaction conditions, kinetics, and specificity of reaction. With this information, rate- and/or yield-limiting reactions can be potentially improved through substitution, synergistic complementation, or protein engineering. Given the wide range of substrates and methods used for GH characterization, it is difficult to compare results across a myriad of approaches to identify high performance and synergistic combinations of enzymes. Here, we describe a platform for systematic screening of GH activities using automatic biomass handling, bioconjugate chemistry, robotic liquid handling, and nanostructure-initiator mass spectrometry (NIMS). Twelve well-characterized substrates spanning the types of glycosidic linkages found in plant cell walls are included in the experimental workflow. To test the application of this platform and substrate panel, we studied the reactivity of three engineered cellulases and their synergy of combination across a range of reaction conditions and enzyme concentrations. We anticipate that large-scale screening using the standardized platform and substrates will generate critical datasets to enable direct comparison of enzyme activities for cocktail design.

## Introduction

Lignocellulosic biomass (Carroll and Somerville, 2009) is a renewable source of energy, capable of providing the nation with clean, renewable transportation fuels. To convert biomass into biofuels, one key step is enzymatic saccharification, which is known to be inefficient and expensive (Klein-Marcuschamer et al., 2012). Thus, low-cost, robust, high-performance enzymes or enzyme cocktails are needed to reduce the overall cost of biofuel production. There are many sources of inedible plant biomass that can serve as feedstocks for biofuel production, including agricultural wastes [corn stover, switchgrass (SG), wood trimming], municipal solid wastes, and emerging bioenergy crops. However, these various feedstocks have different glycan composition, bond linkages, and individual sugar contents, which complicates the development of cost-effective saccharification approaches. Moreover, the content and structure of glycans and lignin from the same biomass may respond in different ways to the prerequisite pretreatments (Li et al., 2010). These variations contribute to the observation that there is no universal enzyme or enzyme cocktail for all substrates and biofuels processes.

Given these process constraints, it is important to characterize saccharification enzymes against a wide range of biomass compositions, pretreatments, and processing conditions to generate data that can help enable customized optimal enzyme cocktails for saccharification (Banerjee et al., 2010; Walton et al., 2011). Given the large number of potential combinations of substrate-pretreatment reaction conditions, it is desirable to have a reliable high throughput enzyme assay methods and standardized panels of substrates and conditions.

Currently, several high throughput glycosyl hydrolyze (GH) assays are available to characterize enzyme activity. The majority of these are based on detection of colorimetric or fluorescent products. For example, the 2,4-dinitrosalicyclic acid (DNS) reducing sugar assay (Decker et al., 2009) can provide rapid analysis of large enzyme libraries. However, it is a non-specific method that can only provide total reducing sugar content. Fluorescence-based enzyme assays using surrogate substrates, e.g., 4-methylumbelliferyl-β-glucopyranoside (van Tilbeurgh et al., 1982) are also available for the evaluation of β glycosidases, are also available. Surrogate substrate methods require preparation of each substrate for each enzyme type, which may be laborious, and the reactivity may be biased versus the native substrate. Moreover, care is required to avoid interference from background absorbance/fluorescence.

To address some of these limitations, we developed a mass spectrometry-based enzyme assay platform called Nimzyme (Northen et al., 2008; Reindl et al., 2011; Greving et al., 2012). The first-generation Nimzyme platform was based on soluble model substrates, which were synthesized chemically (Deng et al., 2012). This approach provided the specificity, sensitivity, and high throughput needed to screen a GH1 library of 175 enzymes. Several high-performance enzymes were identified with desired bioprocessing conditions (70°C, 20% ionic liquid) (Heins et al., 2014). Another manuscript in this volume reports use of the first-generation Nimzyme platform for numerical analysis of reactions with cellotetraose-nanostructure-initiator mass spectrometry (NIMS) (Deng et al., 2014). Results of this work demonstrate diagnostic behaviors of several classes of GH enzymes.

However, the use surrogate substrates does not allow interrogation of the myriad of bonding types present in plant biomass or the three-dimensional arrangements of these bonds present on the plant biomass, and so does not represent a fully realistic approach for the study of enzyme reactions.

To overcome this limitation, we developed an oxime-Nimzyme probe (Deng et al., 2014) to directly study enzyme hydrolysis of plant biomass. In this approach, soluble oligosaccharide products are captured in a stable oxime linkage and then delivered to the NIMS chip for subsequent analysis, while inclusion of ^13^C-labeled monosaccharide standards (glucose and xylose) allows quantitation of the derivatized glycans. Besides the diagnostic detection of solubilized products, this next-generation Nimzyme approach also allows quantitative studies of the time-dependence of product formation, and dissection of individual apparent rates for reactions of individual enzymes with plant biomass.

To further advance application of the Nimzyme approach, here we report the development of a process platform and a panel of 12 diverse glycan substrates. These substrates were selected to represent the diversity of plant glycosidic bond linkages and the sugar compositions that are relevant to biofuel production. We use this panel of substrates to characterize three previously described enzymes from *Clostridium thermocellum* that have been engineered to perform outside of their natural cellulosomal location. These are fusions of the catalytic domains of CelA (gene locus Cthe_0269, GH8 endoglucanase family), CelR (gene locus Cthe_0578, GH9 cellotetrahydrolase), and CelE (gene locus Cthe_0797, GH5 endoglucanase family) to the CBM3a domain from scaffoldin protein CipA. The CBM3a domain used in this work comes from the CipA scaffoldin protein from *C. thermocellum*. CBM3a is a well-studied carbohydrate-binding module helps to promote binding of the enzyme onto the polysaccharide, thus plays a key role in promoting the efficient hydrolysis of cellulose (Yaniv et al., 2012). We envision that the standardization and automation of GH assays enabled by this approach will be valuable in providing large datasets of GH performance needed to select enzymes for improved biomass deconstruction.

## Materials and Methods

### Materials

1,4-β-d-cellotetraose ~95% was purchased from Megazyme (Ireland) Cat. No. O-CTE100, Lot No. 130604; 1,4-β-d-xylotetraose ~95% was purchased from Megazyme (Ireland), Cat. No. O-XTE, Lot No. 120204; 1,4-β-d-mannotetraose ~95% was purchased from Megazyme (Ireland), Cat. No. O-MTE, Lot No. 111004; Arabinoxylan was purchased from Megazyme (Ireland), Cat. No. P-WAXYI, Lot No. 120801a; Carob galactomannan was purchased from Megazyme (Ireland) Cat. No. P-GALML, Lot No. 10501b; beechwood xylan was purchased from Sigma-Aldrich (St. Louis, MO, USA), Cat. No. X4252-100G, Lot No. BCBL2915V.

Switchgrass has emerged as a potential bioenergy crop because it is perennial, resource-efficient, and requires low-inputs for maintenance (Keshwani and Cheng, 2009). Untreated switchgrass (UT-SG), also named as Putnam SG, was obtained from Daniel Putnam at UC Davis; diluted acid pretreated switchgrass (DA-SG) was obtained by mixing Putnam SG with 1% sulfuric acid at 190°C for 0.5 min at NREL. Ammonium fiber expansion pretreated switchgrass (AFEX-SG) was prepared at Michigan State University. Ionic liquid pretreated switchgrass (IL-SG) was prepared at 140°C for 3 h with 15% solid loading in [EMIM][OAc] (1-ethyl-3-methylimidazolium acetate) at JBEI. All four SG substrates (IL-SG, AFEX-SG, DA-SG, and UT-SG) were milled by Thomas Wiley Mill (Model 3383 L1) for 20 min and sieved (passage through a 20-mesh sieve and retention by an 80-mesh sieve). The particle size was found in the range of 200–450 μm. Avicel PH-101 cellulose was requested from FMC Biopolymer (Philadelphia, PA, USA), Lot No. P112824596. Phosphoric acid swollen cellulose (PASC) was prepared from Avicel PH-101 cellulose (FMC Biopolymer) with 85% phosphoric acid as reported (Zhang et al., 2006).

### Synthesis

The synthesis of *O*-alkyloxyamine fluorous tag has been reported previously (Deng et al., 2014).

### Enzymes

The catalytic domains of the enzymes studied were obtained from the following gene loci: CelA (Cthe_0269); CelR (Cthe_0578); and CelE (Cthe_0797). Each of these catalytic domains was fused to the CBM3a domain from the scaffoldin CipA (Cthe_3077). Additional information on these genes can be found at Uniprot (Apweiler et al., 2011). All genes were prepared by PCR using *C. thermocellum* ATCC 27405 genomic DNA as template and cloned into the *Escherichia coli* expression vector pEC_CBM3a to create enzyme_CBM3a fusion proteins, e.g., CelAcc_CBM3a. The vector pEC_CBM3a is a hybrid of pEU_HSBC_CBM3a and pVP65K (Takasuka et al., 2014; Aceti et al., 2015) that yields fusion proteins having an N-terminal enzyme catalytic domain fused by an ~40 aa linker sequence to the CBM3a domain from Cthe_3077. Methods for PCR amplification, capture, and sequence verification of protein coding sequences, transformation into E. coli 10G competent cells (Lucigen, Middleton, WI, USA) for DNA manipulations and E. coli B834 for protein expression were as previously reported (Takasuka et al., 2014). Additional details of the properties and methods for use of pEU is described elsewhere (Aceti et al., 2015).

### Enzyme Plate Construction

Three enzymes (CelAcc-CBM3a, CelRcc-CBM3a, and CelEcc-CBM3a) were chosen to construct an enzyme plate with varied enzyme concentrations (microgram per microliter) shown in Table 1. Each enzyme was present in triplicate as shown for CelAcc-CBM3a (columns A, B, C), CelRcc-CBM3a (columns D, E, F), and CelEcc-CBM3a (columns G, H, I). Some enzyme combinations were also included to investigate the potential synergy among these three enzymes as shown in columns J, K, L.

**Table 1:** **Enzyme plate construction**.

		CelAcc-CBM3a	CelAcc-CBM3a	CelAcc-CBM3a	CelRcc-CBM3a	CelRcc-CBM3a	CelRcc-CBM3a	CelEcc-CBM3a	CelEcc-CBM3a	CelEcc-CBM3a	CelAcc-CBM3a + CelRcc-CBM3a	CelRcc-CBM3a + CelEccCBM3a	CelAcc-CBM3a + CelEcc-CBM3a
Unit		A	B	C	D	E	F	G	H	I	J	K	L
μg/μL	1	0.01	0.01	0.01	0.01	0.01	0.01	0.01	0.01	0.01	0.005 + 0.005	0.005 + 0.005	0.005 + 0.005
μg/μL	2	0.05	0.05	0.05	0.05	0.05	0.05	0.05	0.05	0.05	0.025 + 0.025	0.025 + 0.025	0.025 + 0.025
μg/μL	3	0.1	0.1	0.1	0.1	0.1	0.1	0.1	0.1	0.1	0.05 + 0.05	0.05 + 0.05	0.05 + 0.05
μg/μL	4	0.25	0.25	0.25	0.25	0.25	0.25	0.25	0.25	0.25	0.125 + 0.125	0.125 + 0.125	0.125 + 0.125
μg/μL	5	0.5	0.5	0.5	0.5	0.5	0.5	0.5	0.5	0.5	0.25 + 0.25	0.25 + 0.25	0.25 + 0.25
μg/μL	6	1.25	1.25	1.25	1.25	1.25	1.25	1.25	1.25	1.25	0.625 + 0.625	0.625 + 0.625	0.625 + 0.625
μg/μL	7	2.5	2.5	2.5	2.5	2.5	2.5	2.5	2.5	2.5	1.25 + 1.25	1.25 + 1.25	1.25 + 1.25
μg/μL	8	5	5	5	5	5	5	5	5	5	2.5 + 2.5	2.5 + 2.5	2.5 + 2.5

### Procedures for Handling Insoluble Substrates with Labman Solids-Handling Robot

To minimize static electricity, all plastic labware was prophylactically treated using a Tabletop Ionizing Transport System Model IT-7000 (Electrostatics Incorporated, 90610-03610, Harleysville, PA, USA). Commercially sourced substrates – Avicel PH-101, arabinoxylan, carob galactomannan, and beechwood xylan – were received as fine powders and aliquoted without further processing. The Labman (North Yorkshire, UK) solids-handling robot at JBEI was used to aliquot insoluble substrates (Table 2, numbers 4 through 12) from 2-mL Sarstedt vials (72.694.007) into 340-μL 96-well thermal cycler plates (plate: VWR 82006-636, holder: Axygen R-96-PCR-FY) with a target mass of 2 ± 0.25 mg per well. Feeder vibration power was set to 40% for all substrates and feeding durations were optimized for each to minimize overfeeding. Average feeding durations ranged from 3 to 5 h per 96-well plate depending on the substrate. After aliquoting, substrate plates were sealed using peelable heat seal (Agilent 24210-001) using an Agilent PlateLoc heat sealer set to 175°C for 2.5 s and stored at 4°C.

**Table 2:** **Standardized substrates list**.

Soluble substrates	Insoluble solid substrates
(1) Cellotetraose	(4) Acid swollen cellulose (PASC)
	(5) Avicel PH-101 cellulose
	(6) Arabinoxylan
(2) Xylotetraose	(7) Carob galactomannan
	(8) Beechwood xylan
	(9) IL-switchgrass
(3) Mannotetraose	(10) AFEX-switchgrass
	(11) DA-switchgrass
	(12) UT-switchgrass

*(1) Soluble substrates 1–3 can be dissolved in water and bypass the solid dispersion step by Labman. (2) Insoluble solid substrates 4–12 are being dispersed into a 96-well PCR plate by Labman, then Biomek was used for liquid handling*.

### Procedures for Handling Insoluble Substrates with Biomek FX Robot

For all nine insoluble solid substrates, all the liquid handling for enzymatic hydrolysis reactions and bioconjugation chemistry were performed using a Biomek FX robot equipped with an AP96 multichannel pod (Beckman Coulter). For the enzymatic hydrolysis step, the Biomek FX transferred 180 μL of 50 mM phosphate buffer, pH 6.0, into 96-well PCR plates containing ~2 mg solid substrate that was previously aliquoted using the Labman robot (plate A). Then 20 μL of enzyme solution was transferred from the enzyme plate into plate A. After sealing the 96-well plate with PlateLoc peelable seal (2.5 s, 175°C), the plate was incubated at 60°C for 18 h in a shaker with shaker speed set to 200 rpm (HT INFORS). The recipe for a typical NIMS bioconjugation solution was a mixture of the following: (1) 1 mL of probe solution (150 mM in 1:1 (v/v) H_2_O:MeOH); (2) 6 mL of 100 mM glycine buffer, pH 1.3; 3) 0.5 mL of 5 mM ^13^C glucose aqueous solution; (4) 0.5 mL of 5 mM ^13^C xylose aqueous solution; (5) 2 mL of acetonitrile; (6) 1 mL of methanol. Plate B is a 96-well PCR plate prepared by the Biomek robot containing with 22.2 μL of NIMS tagging solution in each well. After plate A (enzymatic reaction) was cooled to room temperature, a 4 μL aliquot from plate A was transferred into plate B by the Biomek robot. Plate B was sealed with PlateLoc peelable heat seal (2.5 s, 175°C) and left at room temperature for 16 h. Samples (12 μL) from plate B was transferred into the assay plate (Greiner bio-one, 384 well μ clear-plate, coc black, Lo base, 10 pcs/bag, Lot No. E11060DN; Cat. No. 788876, Made in Germany).

### Procedures for Handling Soluble Substrates with Biomek FX Robot

For the three soluble substrates, 1,4-β-d-cellotetraose (G4), 1,4-β-d-xylotetraose (X4), and 1,4-β-d-mannotetraose (M4), 10 mM aqueous solutions were prepared as stock solutions. The Biomek robot was used to transfer 40 μL of 50 mM phosphate, pH 6.0, into each well of a 96-well PCR plate (plate A). Then 5 μL of soluble substrates [G4 (5 mM), X4 (5 mM), or M4 (5 mM)] was transferred to plate A. After that, Biomek transferred 5 μL of enzymes from the enzyme plate to plate A. After sealing the 96-well plate with PlateLoc peelable heat seal (2.5 s, 175°C), the whole plate was incubated at 60°C for 18 h with 200 rpm in a shaker (HT INFORS). After this step, the Biomek robot was used to perform all subsequent liquid-handling steps as indicated above for insoluble substrates.

### Acoustic Printing of Sample Arrays

Samples from the 384-well Greiner plate (1 μL) were acoustically transferred by ATS Acoustic Liquid Dispenser (EDC Biosystems) onto a 2 × 2 inch NIMS chip. Individual reaction spots on the NIMS chip were ionized by a laser and products were detected by a time-of-flight mass spectrometer (TOF/TOF 5800 MALDI systems, AB Sciex).

### Nanostructure-Initiator Mass Spectrometry Imaging

The 4800 imaging acquisition software was used in these experiments. Chips were loaded using a modified standard MALDI plate. Instrument was set with laser intensity at 2,550 and 15 shots per sub-spectrum. The detector voltage multiplier was set as 0.77. For imaging, the step size was set up at 50 μm for both *x* and *y* direction.

### Mass Spectrometry Imaging Data Processing (Open MSI)

The imaging file was uploaded to openmsi.nersc.gov by Globus Connect Personal. The converted file was analyzed by a draggable points notebook written in Python. Signal intensities were identified for the ions of the tagging products. Enzyme activities were determined by measuring the concentration of glycan products using either [*U*]-^13^C glucose or [*U*]-^13^C xylose as an internal standard.

## Results and Discussion

### Substrate Panel

Table 2 lists some of the properties of the substrates included in the substrate panel. More detailed rationale for selection of individual substrates is described in the following experimental sections. The following general principles were used to assemble the substrate panel: (1) substrates should be readily available; (2) some substrates should have known structures (e.g., cellotetraose, xylotetraose, mannotetraose) so that enzyme specificity can be rapidly determined; (3) some substrates should be plant biomass substrates such as SG; and (4) examples of different pretreatments of the same biomass should be included. Based on evolving needs, other substrates can be added to the panel using similar principles.

After selection of these 12 substrates, we sourced large quantities of each substrate so as to permit detailed analytical characterizations and extended experimentation. Each of the substrates was acquired and then divided such that our two institutions (GLBRC and JBEI) each had large aliquots of each substrate.

### Enzymes Selected for Platform Testing

To test this assay platform, we used catalytic domains from three *C. thermocellum* enzymes. In earlier studies (Deng et al., 2014; Takasuka et al., 2014), we showed that CelAcc, CelRcc, and CelEcc were able to release a variety of oligosaccharide products from IL- and AFEX-pretreated SG. As these enzymes are normally included in the cellulosome, we removed the dockerin domains and instead fused them to CBM3a (Yaniv et al., 2012). This engineered addition of CBM3a targets the catalytic domain to a polysaccharide surface. Our studies also showed that CelEcc was a multifunctional GH5 catalytic domain that was reacted with cellulose, xylan, and mannan (classified as CMX). This broad specificity of reaction provides opportunities to understand contributions of an identical active site to biomass hydrolysis. By contrast, CelRcc_CBM3a reacted only with cellulose (classified as C), while CelAcc_CBM3a reacted with cellulose and only weakly with xylan (classified as CX), and so provided more specific enzyme reactions.

### Automated Enzyme Assay Platform

**Figure 1:**
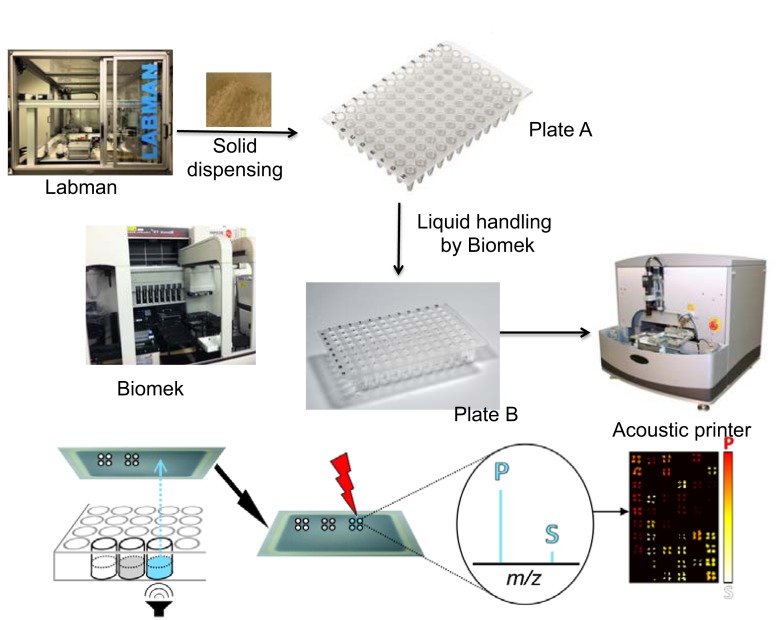
**Workflow of oxime-NIMS automation developed to study GHs**. (1) Solid biomass was dispersed by Labman. (2) Liquid handling was performed by Biomek Automation Workstation, including setup of enzyme and oxime bioconjugation reactions. (3) Sample array on the NIMS chip was generated by an acoustic printer (ATS Acoustic Liquid Dispenser). (4) Mass spectrometry imaging (MSI) provided the readout of experimental assay results.

Efforts have been made to establish a simple automated workflow that enables high-throughput and that minimizes assay error. In a preparatory step (Figure 1), insoluble substrates (Table 2, 4 through 12) were aliquoted into 96-well thermal cycler plates for activity assays using a Labman solids-handling robot and stored at 4°C until use. Immediately before initiating the activity assays, enzymes were manually aliquoted into 96-well plates.

The individual steps of the workflow are as follows. In step 1, reactions were prepared using a Biomek FX liquid-handling robot to add enzyme to substrate. In step 2, reactions were incubated at 60°C for 18 h with shaking. In step 3, an aliquot from the reaction plate was combined with the oxime-NIMS tagging solution using the liquid-handling robot, after which the NIMS tag reacted with reducing sugar to form a stable oxime linkage during overnight incubation. In step 4, the solution containing NIMS-tagged glycans was transferred from tagging reactions onto a NIMS chip mass spectrometry surface by sequentially using (1) the liquid-handling robot to reformat aliquots from the reactions in 96-well plates into 384-well plates followed by (2) using an ATS Acoustic Liquid Dispenser to print 1-nL droplets onto the chip via non-contact dispensing. In step 5, an AB SCIEX TOF/TOF 5800 mass spectrometer was used to image the NIMS chip to collect data on the tagged glycans. In step 6, the data were processed using OpenMSI (a free web-based visualization, analysis and management package for mass spectrometry imaging (MSI) data) to quantify the tagged glycans in order to compute enzyme activities.

The different characteristics of the substrates introduced challenges for automated sample handling. Thus, while soluble samples can be dispensed using standard liquid handling (Figure 1), insoluble samples were more challenging and required application of solid dispensing using a Labman system. As shown in Table 2, only 3 out of the 12 substrates were soluble and could processed using simple liquid handling, whereas the nine other substrates were insoluble and required Labman to carry out relatively slow protocol to generate batches of substrate-filled 96-wells PCR plates that were then stored at −20°C freezer until required.

### Reactions with Soluble Substrates

Cellotetraose (G4), xylotetraose (X4), and mannotetraose (M4) are purified oligosaccharides that can be used to study cellulase, xylanase, and mannase activities, respectively. These soluble substrates also permit simple liquid-handling approaches. However, it is important to recognize that some GH enzymes require longer oligosaccharide chains to show effective catalysis.

The reactions of G4, X4, and M4 were screened using a 96-well enzyme plate containing individual wells of CelAcc_CBM3a, CelRcc_CBM3a, and CelEcc_CBM3a, and mixtures of these three (Table 1). All three endoglucanases produced cellobiose as the major product from G4 under various concentrations (Tables S1–S3 in Supplementary Material). No products were observed at the lowest enzyme concentration tested (1 ng/μL) for all three enzymes. When the enzyme concentration was increased (5 ng/μL), CelEcc_CBM3a gave complete hydrolysis of the cellotetraose present into smaller oligosaccharides, while CelRcc_CBM3a hydrolyzed about 80% of G4 and CelAcc_CBM3a hydrolyzed about 13% of G4. For CelAcc_CBM3a, increasing the enzyme concentration above 0.01 μg/μL was needed to obtain complete hydrolysis. Thus, the concentration screening can give a preliminary assessment of the affinity of an enzyme for the oligosaccharide. In the hydrolysis of cellotetraose, the following effective concentrations of enzyme were determined: CelAcc_CBM3a (10 ng/μL), CelRcc_CBM3a (10 ng/μL), and CelEcc_CBM3a (5 ng/μL).

Xylotetraose (X4) is an oligosaccharide with β 1,4-linked xylose unit, and cellulases CelAcc_CBM3a and CelRcc_CBM3a were not reactive with xylotetraose. However, the multifunctional enzyme CelEcc_CBM3a was active with xylotetraose. Increasing the enzyme concentration increased oligomer and monomer products (cellotriose, cellobiose, and xylose, Table S4 in Supplementary Material). For CelEcc_CBM3a with xylotetraose, the minimum amount of enzyme needed for complete hydrolysis of X4 was 0.25 μg/μL. This is a 50-fold increase in the amount of enzyme needed to hydrolyze xylotetraose versus cellotetraose, suggesting higher affinity for the hexose oligosaccharide.

None of the enzymes studied reacted with mannotetraose (M4). Since CelAcc_CBM3a and CelRcc_CBM3a are cellulases, this was not surprising. However, since CelEcc_CBM3a has been shown to react with mannan and glucomannan, the lack of reaction with M4 was not expected. This may arise from the possibility that mannotetraose is not long enough to bind effectively in the catalytic channel of CelEcc_CBM3a.

### Reactions with Avicel and PASC

**Figure 2:**
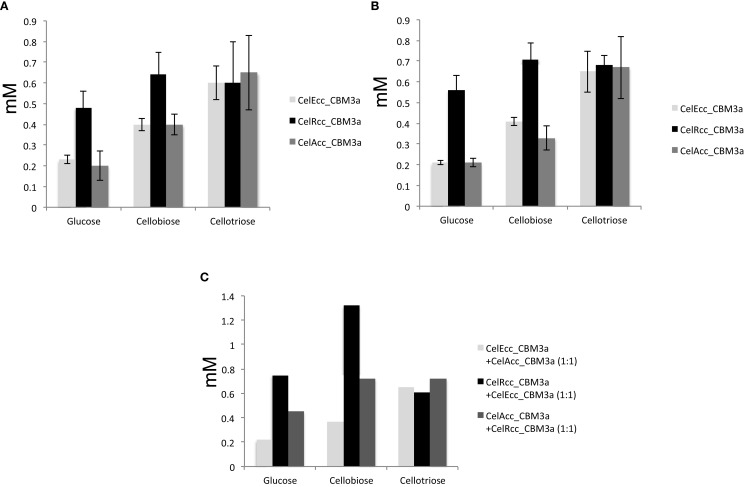
**Synergistic effect of CelRcc_CBM3a + CelEcc_CBM3a: (A)** Enzyme loading of 25 mg/g Avicel for CelEcc-CBM3a, CelRcc-CBM3a, and CelAcc-CBM3a, respectively; **(B)** Enzyme loading of 50 mg/g Avicel for CelEcc-CBM3a, CelRcc-CBM3a, and CelAcc-CBM3a, respectively; **(C)** Total enzyme loading are 50 mg/g Avicel for CelEcc-CBM3a + CelAcc-CBM3a, CelRcc_CBM3a + CelEcc_CBM3a, and CelAcc_CBM3a + CelRcc_CBM3a, respectively.

Phosphoric acid swollen cellulose (Zhang et al., 2006) has become a very popular substrate to study cellulase activities because it has a relatively easily hydrolyzed amorphous habit (Sharrock, 1988; Wood, 1988; Wood and Bhat, 1988). Solid-state cross-polarization magic angle spinning ^13^C NMR was used to determine the crystallinity (Park et al., 2010) of both Avicel and PASC used in this work. The NMR results demonstrated that Avicel had crystallinity of 53% while PASC had crystallinity of less than 5% (Figure S1 in Supplementary Material). Compared with the microcrystalline cellulose (Avicel), amorphous PASC has the advantages of practical solubility and increased accessibility to the cellulases. Therefore, it is more easily hydrolyzed by cellulases. As expected, all three cellulase enzymes produced multiple times more soluble hexose products from PASC than Avicel (4 times for CelEcc_CBM3a, 2.5 times for CelRcc_CBM3a, and 3 times more for CelAcc_CBM3a) (Tables S5 and S6 in Supplementary Material). For the hydrolysis of Avicel, CelRcc_CBM3a performed better than CelEcc_CBM3a and CelAcc_CBM3a. Interestingly, the enzyme combination of CelEcc_CBM3a and CelRcc_CBM3a worked better than the combination of either CelAcc_CBM3a and CelRcc_CBM3a or CelEcc_CBM3a and CelAcc_CBM3a (Figure 2). Since the amount of glycan products produced by the combination of CelEcc_CBM3a and CelRcc_CBM3a was greater than the scaled contributions of the individual enzymes, this combination is demonstrated to have a synergistic effect in reaction.

### Reactions with Beechwood Xylan and Arabinoxylan

Beechwood xylan has a high xylose content (~84%) with a majority of β-1,4 linkages. Arabinoxylan consists of a mixture of arabinose and xylose in an approximate 40:60 ratio with β-1,4- and β-1,6-linkages. Our screening results showed that CelRcc_CBM3a was unable to hydrolyze either beechwood xylan or arabinoxylan, even at high enzyme loading. By contrast, CelAcc_CBM3a showed weak activity with beechwood xylan, and at the highest enzyme loading tested (50 mg/g xylan), about 20% of the xylan was hydrolyzed (Table S8 in Supplementary Material). Furthermore, CelEcc_CBM3a reacted well with beechwood xylan, and at the highest enzyme concentration tested (50 mg/g xylan), about 40% of the xylan was hydrolyzed into soluble pentose products. Both CelAcc_CBM3a and CelEcc_CBM3a had only weak activities with arabinoxylan, even at the highest enzyme loading tested (50 mg/g xylan).

### Reactions with Galactomannan

Galactomannans are polysaccharides consisting of a mannose backbone with galactose side groups. Mannans are important constituent of hemicellulose in some plant biomass. (Malherbe et al., 2014) For example, softwoods contain 15–20% (w/w) mannans (Rodríguez-Gacio et al., 2012) and legume seeds can contain more than 30% of mannans (Buckeridge, 2010). For hydrolysis of these types of biomass into simple monosaccharides, it is important to find enzymes that can efficiently degrade mannans. Consequently, galactomannan was included in the substrate panel to test for mannanase activities. The screening results show that cellulases (CelAcc_CBM3a and CelRcc_CBM3a) could not deconstruct galactomannan at all. However, CelEcc_CBM3a (Table S9 in Supplementary Material) was able to hydrolyze galactomannan to hexose products (Fox et al., 2014).

It is interesting to compare the results of CelEcc_CBM3a reaction with galactomannan and its lack of reaction with mannotetraose. Besides the length of the oligosaccharide suggested above, it is also plausible that CelEcc_CBM3a may prefer reaction with the galactose-substituted mannans.

### Reactions with Switchgrass

The structural features of SG, including surface area, crystallinity, the contents of cellulose, hemicellulose, and lignin vary considerably depending on pretreatment (Xu and Huang, 2014). For example, cellulose is changed from cellulose I (untreated) to cellulose II after ionic liquid pretreatment (Cui et al., 2014). Other factors, like degree of delignification, hemicellulose solubilization (especially in diluted acid pretreatment), changes in porosity, and others affect enzyme accessibility so that different glycan products are produced for different pretreated SG. The compositional analysis of these four pretreated samples demonstrates these significant differences caused by pretreatment (Table S10 in Supplementary Material). These compositional differences imply the need for customized enzyme cocktails.

Four types of pretreated SG were included in the substrate panel to permit comparative studies of the consequences of pretreatment on enzymatic saccharification. These are UT-SG as control, IL-SG (Li et al., 2010), AFEX-SG (Bals et al., 2010), and DA-SG (Pu et al., 2013). This selection covers the three predominant pretreatment methods under investigation by the US-DOE funded Bioenergy Research Centers (Singh et al., 2015).

Screening results show that neither the three individual enzymes (CelAcc_CBM3a, CelRcc_CBM3a, and CelEcc_CBM3a) nor their combinations could deconstruct UT-SG. This is consistent with the substantial value of pretreatment before enzymatic saccharification.

For DA-SG, all assays across the breadth of enzyme concentrations gave similar, low yields for both hexose and pentose products. The relatively low reactivity of these individual enzymes with crystalline cellulose is consistent with the low yield of hexose product. Presumably, the low amount of xylan remaining in DA-SG (4.58%) is not easily accessible to further enzyme hydrolysis. For example, although CelEcc_CBM3a reacts well with pure xylan and the hemicellulose fractions in AFEX-SG and IL-SG (see below), it did not react with the remaining xylan in DA-SG. Whether this is a result of depletion of reactive substructures or other aspects of the diluted acid pretreatment is not clear.

For reactions with AFEX-SG, both CelRcc_CBM3a and CelAcc_CBM3a produced little soluble glycan and no soluble pentose. By contrast, CelEcc_CBM3a performed much better, and yielded both hexose and pentose products. CelEcc_CBM3a was especially reactive with the hemicellulose portion of AFEX-SG, yielding about 2.5× more pentose products than in its reaction with IL-SG (Table S11 in Supplementary Material).

**Figure 3:**
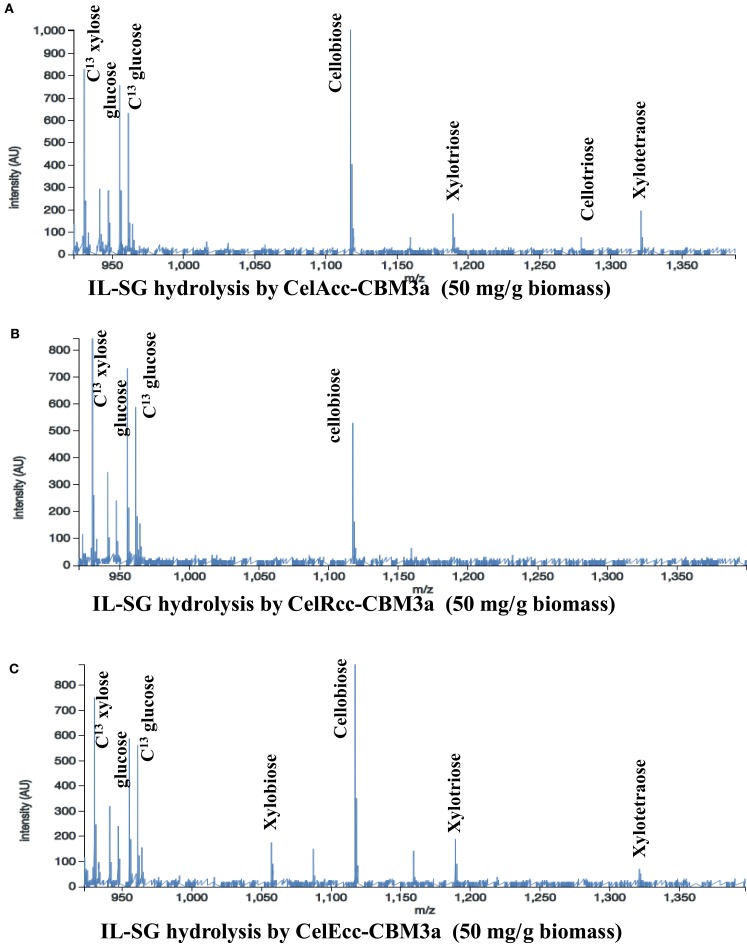
**Mass spectra from OpenMSI data processing**: Reactions of IL-SG with CelAcc_CBM3a **(A)**, CelRcc_CBM3a **(B)**, and CelEcc_CBM3a **(C)**.

For IL-SG, all three enzymes (Figure 3) produce significant amount of hexose products (Table S12 in Supplementary Material), which can be attributed to the ability of the IL pretreatment to reduce the crystallinity of cellulose. CelAcc_CBM3a produced the most hexose products among these three enzymes, and under the highest enzyme loading of 50 mg/g biomass a conversion of 24% of the glycan was observed. A comparison of the reaction of CelEcc_CBM3a with either IL-SG or AFEX-SG under the same experimental conditions (enzyme loading of 50 mg/g biomass) showed that CelEcc_CBM3a produced 8× more of hexose products with IL-SG than AFEX-SG. This result from the automated platform matches the conclusion that cellulose in IL-SG is much easier to be accessed and digested by CelEcc_CBM3a obtained in earlier oxime-NIMS studies (Deng et al., 2014).

## Conclusion

In this work, we described a panel of 12 substrates and automated platform for characterization of GH enzymes using the oxime-NIMS approach. Standardization is an important step toward more in-depth comparison of GH enzymes activities, both from natural environments and from engineered systems. These studies are consistent with our earlier assignment that CelEcc_CBM3a is a multifunctional enzyme that has cellulase, mannanase, and hemicellulase activities. By contrast, CelAcc_CBM3a has cellulase and only weak hemicellulase while CelRcc-CBM3a only has cellulase activity. This platform automates the handling of both solid biomass and soluble substrates, the introduction of enzymes as individuals or combinations, and the recovery of products for high sensitivity and high-resolution mass spectral analysis. Simplex optimization of the ratios of natural or engineered enzyme combinations produced by systems biology approaches such as gene synthesis and robotic cell-free translation, as well as optimization of the pretreatment conditions can be readily undertaken using this platform.

## Author Contributions

KD, BF, and TN designed experiments. KD, JMG, JG, HT, VR-O, XC, NS, RH, TT, LB, HG-H, and SD carried out experimental work, KD, BB, BF, and TN analyzed results, and KD, JMG, BF, and TN prepared the manuscript. DL, KS, BS, and PA supervised the study. All authors read and approved the final manuscript.

## Conflict of Interest Statement

Kai Deng and Trent R. Northen are co-inventors on a patent application that covers the oxime-NIMS assay. Taichi E. Takasuka and Brian G. Fox are co-inventors on a patent application that covers use of multifunctional enzymes. The remaining authors have no conflict of interest to declare.

## Abbreviations

AFEX-SG, ammonia fiber expansion pretreated switchgrass; CBM, carbohydrate binding module; CelE, broad specificity GH family 5 (GH5) domain from *C. thermocellum* Cthe_0797; DA-SG, diluted acid pretreated switchgrass; GH, glycoside hydrolase; IL-SG, ionic liquid pretreated switchgrass; NIMS, nanostructure-initiator mass spectrometry UT-SG, untreated switchgrass.
