# Supplementary material for: Development of a High Throughput Platform for Screening Glycoside Hydrolases Based on Oxime-NIMS
Source: Front Bioeng Biotechnol. 2015 Oct 13;3:153. doi: 10.3389/fbioe.2015.00153 (PMC4603251; doi:10.3389/fbioe.2015.00153)
Supplement: Supplementary file 1 [file Data_Sheet_1.PDF]

Supplementary Material: Development of a High Throughput Platform for Screening Glycoside Hydrolases based on Oxime-NIMS

Kai Deng,[1,2,*] Joel M. Guenther,[1,2] Jian Gao,[4] Ben Bowen,[4] Huu Tran,[1,2] Vimalier Reyes-Ortiz,[1,4] Xiaoliang Cheng,[1,4] Noppadon Sathitsuksanoh, [1,4] Richard Heins, [1,2] Taichi E. Takasuka,[3] Lai F. Bergeman,[3] Henrik Geertz-Hansen,[1] Samuel Deutsch,[4,5] Dominique Loqué,[1,4] Kenneth L. Sale,[1,2] Blake A. Simmons,[1,2] Paul D. Adams[1,4,6] Anup K. Singh,[1,2] Brian G. Fox,[3,7] Trent R. Northen[1,4,*]

[1]US Department of Energy Joint BioEnergy Institute, Emeryville, CA. [2]Department of Bioengineering and Biotechnology, Sandia National Laboratories, Livermore, CA. [3]US Department of Energy Great Lakes Bioenergy Research Center, University of Wisconsin, Madison, WI. [4]Lawrence Berkeley National Laboratory, Berkeley, CA. [5]Joint Genome Institute, Walnut Creek, CA. [6]Department of Bioengineering, University of California, Berkeley, CA. [7]Department of Biochemistry, University of Wisconsin, WI.

Correspondence

Kai Deng
Joint BioEnergy Institute/
Sandia National Laboratories
5885 Hollis St
Emeryville, CA 94608
kdeng@sandia.gov

Trent R. Northen
Joint BioEnergy Institute
Lawrence Berkeley National Laboratory
5885 Hollis St
Emeryville, CA 94608
trnorthen@lbl.gov

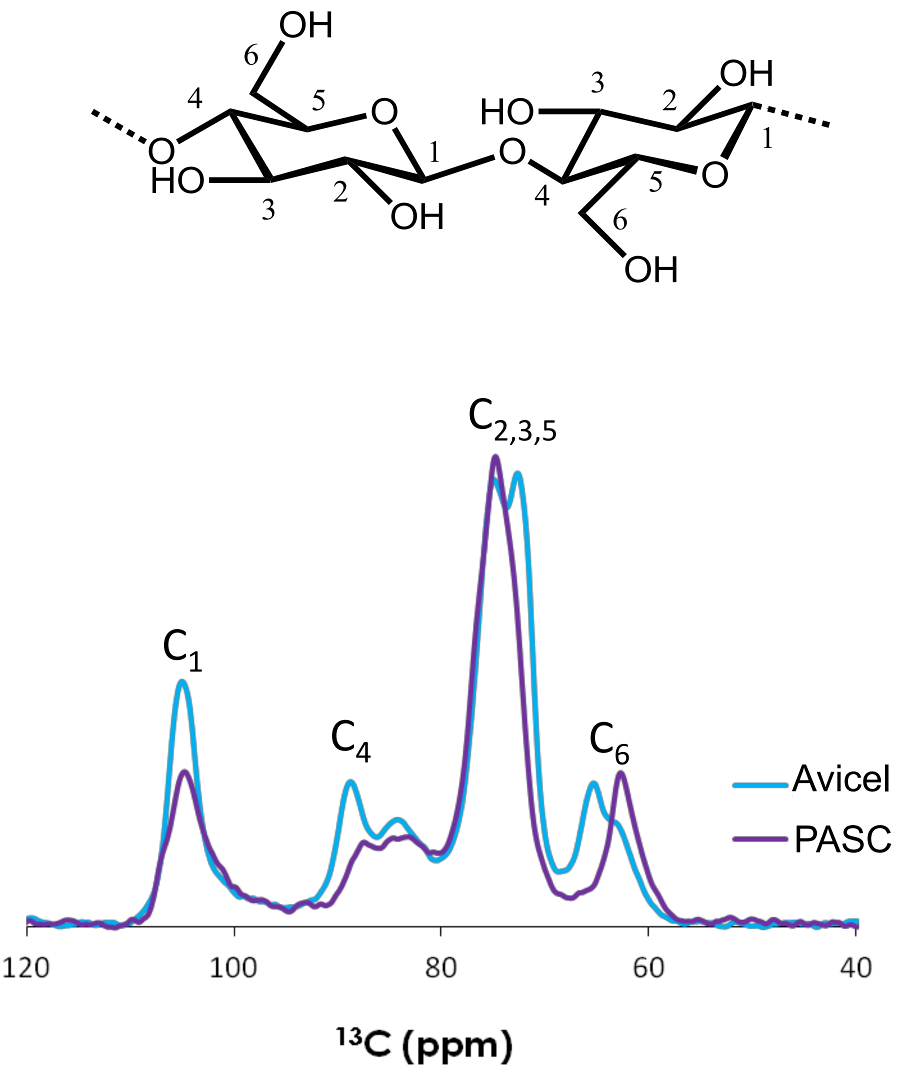

Figure S1 CP/MAS 13C NMR reveals changes in H bonding from Avicel to PASC

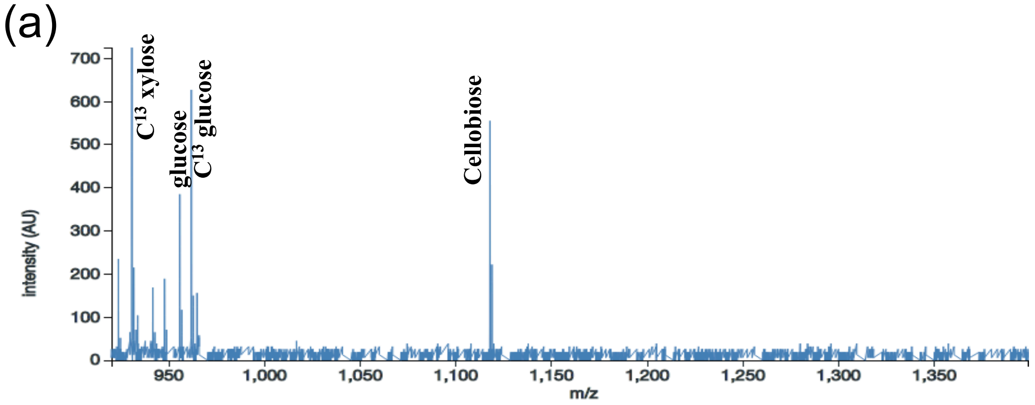

(a) Cellotetraose hydrolysis by CelAcc-CBM3a (25 mg/g biomass)

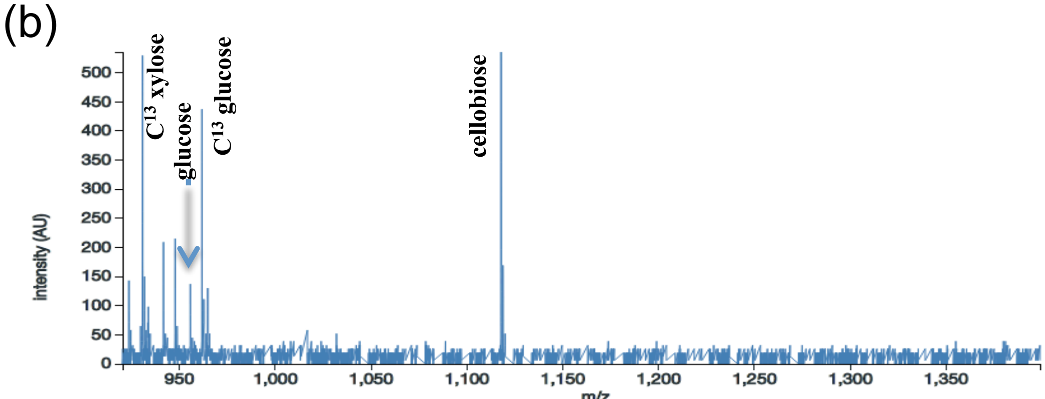

(b) Cellotetraose hydrolysis by CelRcc-CBM3a (25 mg/g biomass)

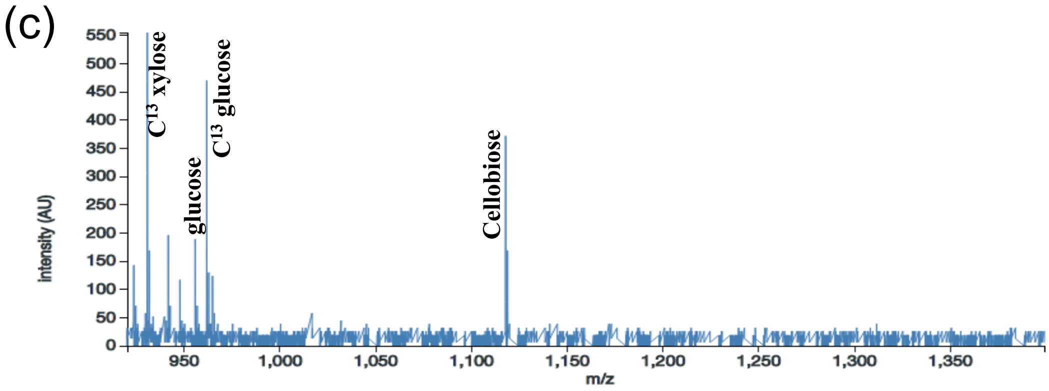

(c) Cellotetraose hydrolysis by CelEcc-CBM3a (25 mg/g biomass)

Figure S2. Mass spectra from OpenMSI data processing. Reactions of cellotetraose with CelAcc_CBM3a (a), CelRcc_CBM3a (b) and CelEcc_CBM3a (c).

Table S1: Glycan products profile from CelEcc-CBM3a with cellotetraose (G4) under various enzyme concentration

Enzyme (ug/ul)	glucose (G1) mM	cellobiose (G2) mM	cellotriose (G3) mM	cellotetraose (G4) mM
0.001	0	0	0	1
0.005	0.21±0.02	1.45±0.16	0.39±0.02	0
0.01	0.31±0.04	1.56±0.04	0.23±0.02	0
0.025	0.29±0.04	1.68±0.06	0.00	0
0.05	0.39±0.03	1.64±0.01	0.00	0
0.125	0.49±0.05	1.64±0.13	0.00	0
0.25	0.53±0.04	1.61±0.06	0.00	0
0.5	0.57±0.08	1.55±0.15	0.00	0

Table S2: Glycan products profile from CelRcc-CBM3a with cellotetraose (G4) under various enzyme concentration

Enzyme (ug/ul)	glucose (G1) mM	cellobiose (G2) mM	cellotriose (G3) mM	cellotetraose (G4) mM
0.001	0	0	0	1
0.005	0.38±0.04	0.77±0.14	0.42±0.05	0.22±0.05
0.01	0.47±0.05	1.21±0.16	0.39±0.04	0.00
0.025	0.65±0.02	1.39±0.05	0.21±0.02	0.00
0.05	0.82±0.08	1.59±0.01	0.00	0.00
0.125	0.52±0.04	1.89±0.24	0.00	0.00
0.25	0.37±0.06	1.77±0.08	0.00	0.00
0.5	0.27±0.03	1.77±0.05	0.00	0.00

Table S3: Glycan products profile from CelAcc-CBM3a with cellotetraose (G4) under various enzyme concentration

Enzyme (ug/ul)	glucose (G1) mM	cellobiose (G2) mM	cellotriose (G3) mM	cellotetraose (G4) mM
0.001	0.00	0.00	0.00	1.00
0.005	0.00	0.22±0.03	0.00	0.87±0.05
0.01	0.51±0.08	0.77±0.11	0.47±0.07	0.00
0.025	0.53±0.02	0.97±0.08	0.42±0.04	0.00
0.05	0.61±0.08	1.15±0.13	0.40±0.04	0.00
0.125	0.74±0.05	1.44±0.07	0.00	0.00
0.25	0.74±0.01	1.44±0.10	0.00	0.00
0.5	0.73±0.03	1.53±0.03	0.00	0.00

Table S4: Glycan products profile from CelEcc-CBM3a with xylotetraose (X4) under various enzyme concentration

Enzyme (ug/ul)	xylose (X1) mM	xylobiose (X2) mM	xylotriose (X3) mM	xylotetraose (X4) mM
0.001	0.16±0.02	0.00	0.00	0.98±0.05
0.005	0.16±0.04	0.00	0.00	0.88±0.02
0.01	0.16±0.03	0.00	0.00	0.84±0.06
0.025	0.15±0.02	0.17±0.01	0.00	0.82±0.03
0.05	0.19±0.03	0.37±0.01	0.00	0.63±0.1
0.125	0.24±0.03	0.76±0.03	0.36±0.06	0.34±0.04
0.25	0.27±0.03	1.04±0.01	0.32±0.02	0.00
0.5	0.32±0.03	1.08±0.13	0.36±0.02	0.00

Table S5 Single Enzyme with PASC

Enzymes (loading 50 mg/g biomass)	Glucose (mM)	Cellobiose (mM)	Cellotriose(mM)
CelEcc-CBM3a	2.60±0.32	4.5±0.42	0.22±0.02
CelRcc_CBM3a	3.16±0.22	3.36±0.18	0.05±0.01
CelAcc-CBM3a	1.75±0.1	3.19±0.13	0.27±0.04

Table S6 Single Enzyme with Avicel

Enzymes (loading 25 mg/g biomass)	Glucose (mM)	Cellobiose (mM)	Cellotriose (mM)	Enzymes (loading 50 mg/g biomass)	Glucose (mM)	Cellobiose (mM)	Cellotriose (mM)
CelEcc-CBM3a	0.23±0.02	0.40±0.03	0.60±0.08	CelEcc-CBM3a	0.21±0.01	0.41±0.02	0.65±0.1
CelRcc_CBM3a	0.48±0.08	0.64±0.11	0.60±0.20	CelRcc_CBM3a	0.56±0.07	0.71±0.08	0.68±0.05
CelAcc-CBM3a	0.20±0.07	0.40±0.05	0.65±0.18	CelAcc-CBM3a	0.21±0.02	0.33±0.06	0.67±0.15

Table S7 Synergistic Effect for CelRcc_CBM3a+CelEcc_CBM3a

Enzyme binary combination	Glucose (mM)	Cellobiose (mM)	Cellotriose (mM)
CelEcc-CBM3a+CelAcc-CBM3a (1:1)	0.22	0.37	0.65
CelRcc_CBM3a+CelEcc-CBM3a (1:1)	0.75	1.32	0.61
CelAcc-CBM3a+CelRcc-CBM3a (1:1)	0.45	0.72	0.72

Total enzyme loading 50 mg/g biomass with Avicel

Table S8 CelAcc-CBM3a and CelEcc-cBM3a (enzyme loading 50 mg/g biomass) with Beechwood Xylans

Enzymes	xylose (X1) mM	xylobiose (X2) mM	xylotriose (X3) mM	xylotetraose (X4) mM
CelAcc-CBM3a	0.11±0.01	0.30±0.05	2.17±0.14	1.30±0.07
CelEcc-CBM3a	0.33±0.05	2.95±0.19	4.04±0.35	1.00±0.12

Table S9 CelEcc-CBM3a with Galactomannan

Enzyme (ug/ul)	glucose (G1) mM	cellobiose (G2) mM	cellotriose (G3) mM
0.001	0.00	0.00	0.00
0.005	0.00	0.00	0.00
0.01	0.00	0.00	0.00
0.025	0.14±0.01	0.20±0.02	0.54±0.05
0.05	0.20±0.05	0.25±0.02	0.79±0.06
0.125	0.20±0.03	0.61±0.07	1.30±0.16
0.25	0.20±0.02	1.15±0.1	1.60±0.12
0.5	0.29±0.04	1.05±0.1	1.56±0.13

Table S10 Composition analysis by Microbac

Sample	%Lignin	%GLucan	%Xylan	%Galactan	%Arabinan	%Mannan	%Water Extractable	%Ethanol Extractives
IL-SG	13.01	50.17	18.23	0	4.77	0	9.49	2.3
AFEX-SG	11.65	40.63	15.43	0	3.84	0	20.38	6.42
DA_SG	17.52	54.74	4.58	0	0	0	6	13.3
UT-SG	18.6	34.12	22.6	1.09	2.67	0.25	7.92	2.9

Table S11 CelEcc-CBM3a 50 mg/biomass

	Glucose (mM)	Cellobiose (mM)	Cellotriose (mM)	Pentose (mM)	Pentobiose (mM)	Pentotriose (mM)	Pentotetraose (mM)
AFEX_SG	0.22±0.03	0.25±0.04	0.00	0.18±0.05	0.82±0.16	1.13±0.11	0.65±0.05
IL_SG	1.08±0.06	1.85±0.08	0.39±0.05	0.14±0.02	0.32±0.07	0.47±0.07	0.21±0.02
DA_SG	0.26±0.04	0.20±0.02	0	0.20±0.04	0	0	0
UT_SG	1.11±0.16	0	0	0.11±0.01	0	0	0

Table S12 Enzyme loading 50 mg/g biomass with IL-SG

	Glucose (mM)	Cellobiose (mM)	Cellotriose (mM)
CelEcc-CBM3a	1.08±0.06	1.85±0.08	0.39±0.05
CelRcc_CBM3a	1.32±0.2	1.43±0.11	0.49±0.10
CelAcc-CBM3a	1.29±0.15	2.23±0.18	0.37±0.02
